# Dual-parameter risk stratification based on device landing zone calcification and aortic annular perimeter for paravalvular regurgitation after self-expanding TAVR

**DOI:** 10.1016/j.ejro.2025.100719

**Published:** 2025-12-10

**Authors:** Jun Shu, Didi Wen, Jingji Xu, Yu Mao, Hui Ma, Jing Zhang, Yao Zhao, Jian Yang, Minwen Zheng

**Affiliations:** a Department of Radiology, Xijing Hospital, Fourth Military Medical University, Changle West Road No. 127, Xi’an City, Shaanxi Province 710032, China; b Department of Cardiovascular Surgery, Xijing Hospital, Fourth Military Medical University, Changle West Road No. 127, Xi’an City, Shaanxi Province 710032, China; c Department of Ultrasound, Xijing Hospital, Fourth Military Medical University, Changle West Road No. 127, Xi’an City, Shaanxi Province 710032, China

**Keywords:** Transcatheter aortic valve replacement, Paravalvular regurgitation, Device landing zone calcification, Aortic annular perimeter

## Abstract

**Purpose:**

The study aimed to identify independent predictors associated with paravalvular regurgitation (PVR) after self-expanding transcatheter aortic valve replacement (SE-TAVR) and to develop a dual-parameter risk stratification model.

**Methods:**

This retrospective study enrolled 292 severe aortic stenosis patients underwent SE-TAVR. PVR severity was assessed pre-discharge. Multivariate logistic regression identified independent predictors of mild/moderate PVR, optimal cutoff values for significant anatomical parameters were determined using receiver operating characteristic (ROC) curve analysis. Patients were subsequently stratified into three risk groups based on these thresholds.

**Results:**

Mild/moderate PVR occurred in 24.0 % of patients. Independent predictors included aortic annular perimeter (OR:1.067, *P* = 0.015), device landing zone calcific volume (OR:1.006 per 10 mm³, *P* = 0.025), and presence of sealing skirt (OR:0.412, *P* = 0.010). The combination of these predictors had a higher discriminative performance (AUC=0.779) than single predictors (*P* = 0.036, 0.007, and <0.001, respectively), with significant integrated discrimination improvement (integrated discrimination improvement=5.4–6.7 %, *P* < 0.001). ROC-derived thresholds (device landing zone calcific volume≥1240 mm³ and aortic annular perimeter≥77 mm) stratified patients into three risk groups with progressively increasing PVR incidence: Group A (neither elevate):8.4 %; Group B (either elevated):23.7 %; and Group C (both elevated):48.7 %. Pairwise comparisons confirming differences between Group A vs. B (*P* = 0.003) and Group B vs. C (*P* < 0.001). Sealing skirts significantly reduced PVR in Groups A (*P* = 0.042) but not in Group B and C (*P* = 0.082 and 0.342).

**Conclusion:**

The dual-parameter model based on device landing zone calcification and aortic annular perimeter significantly enhances PVR risk stratification after SE-TAVR. The dual-threshold model provides a clinically actionable tool for pre-procedural risk stratification and personalized valve selection.

## 1 Introduction

Transcatheter aortic valve replacement (TAVR) has revolutionized the management of severe aortic stenosis (SAS), offering a minimally invasive alternative to surgical intervention [1], [2]. Despite accumulated experience and advancements in transcatheter heart valves (THVs) technology, the occurrence of mild or more paravalvular regurgitation (PVR) remains high and affects more than 40 % of patients [3], [4], [5]. Even mild PVR has been associated with increased late mortality [6], [7], underscoring the importance of addressing this persistent challenge.

THVs are broadly classified into self-expanding (SE) and balloon-expandable (BE) designs, the former showing superior hemodynamic performance especially for patients with small annuli [8], [9], however with a higher incidence of PVR [10], [11], this presenting a significant clinical dilemma. Although a number of PVR predictors have been proposed [5], [12], [13], [14], [15], [16], [17], there is no broad consensus on an optimal strategy in patient or prosthesis selection to reduce PVR.

To address this unmet need, our study aims to identify the parameters independently associated with PVR after SE-TAVR, and to integrate them into a refined dual-parameter risk assessment model. We aim to develop a refined risk assessment model that enhances procedural planning, valve selection, and ultimately, clinical outcomes.

## 2 Materials and methods

### 2.1 Study design and patient cohort

This retrospective cohort study included 374 consecutive patients with SAS who underwent TAVR at XX Hospital between October 1, 2018, and June 30, 2024. The study adhered to the Declaration of Helsinki, with ethical approval from the institutional review board and waived informed consent for retrospective data analysis. Patients were excluded if they had inadequate CT image quality (n = 20), structural valve deterioration (n = 3), valve-in-valve procedures (n = 14), or received BE valves (n = 45). Clinical data were retrieved from electronic medical records.

### 2.2 CT Acquisition and image analysis

We acquired preprocedural cardiac CT angiography (CCTA) using second- or third-generation dual-source CT scanners (Somatom Force/Definition Flash, Siemens Healthineers). Retrospective ECG gating was used to minimize motion artifacts, with scanning parameters adjusted for body mass index (tube voltage: 100–120 kV; tube current: 220 mAs). Contrast agent (Ultravist 370 mg/mL) was administered at 4–6 mL/s, followed by a saline flush.

A radiologist with 10 years of experience in cardiovascular imaging analyzed images using 3D Slicer (v5.0.3) with Custom Python scripts. The device landing zone, defined as the aortic valve complex (from the virtual annulus to coronary ostia) and left ventricular outflow tract (10 mm inferior to the annulus), was segmented in diastolic phase images (usually 70 % R-R interval). Gaussian mixture modeling was applied to quantify calcific volume by distinguishing calcified tissue from blood pool (HU threshold: upper 99.7 % of blood pool values) [18], [19], [20], with details provided in Supplementary Figure 1. As the sizing strategy of SE-TAVR was guided by the perimeter as the determined factor [21], [22], aortic annulus perimeter was measured using manual contour tracing in systolic phase images (usually 30 % R-R interval) in this study, with relative percent oversizing calculated as: THV perimeter oversizing (%) = (THV nominal perimeter/annular perimeter – 1) × 100. Ellipticity index (%) was defined as: (maximum annular diameter / minimum annular diameter) × 100. The bicuspid aortic valve (BAV) and tricuspid aortic valve (TAV) phenotypes were determined by MSCT. BAV morphologies were defined according to the Sievers terminology [23].

To assess the interobserver repeatability of device landing zone calcific volume and aortic annulus perimeter measurements, 60 randomly selected cases were independently reanalyzed by a second radiologist with 5 years of experience in cardiovascular imaging, using the same methodology. This radiologist was blinded to the initial analysis results.

Additionally, the location (annulus, LVOT, and leaflets) and severity of calcifications were semi-quantitatively graded as none, mild, moderate, or severe [24] by both radiologists independently. Representative examples are provided in Supplementary Figure 2. Any discrepancies between the two readers were resolved through consensus discussion.

### 2.3 Echocardiographic evaluation

Preoperative transthoracic echocardiography (TTE) data were obtained according to recommendations of echocardiographic societies [25], and included left ventricular ejection fraction (LVEF), peak aortic valve velocity, mean aortic valve gradient by modified Bernoulli formula, aortic valve area calculated using the continuity equation.

A certified echocardiographer with 12 years of clinical experience in the echocardiographic evaluation of prosthetic heart valves performed all pre-discharge transthoracic echocardiographic assessments. PVR was graded using an integrative multi-parametric approach, which included visual assessment of the number of regurgitant jets, jet width at origin, and circumferential extent. Grading was performed according to Valve Academic Research Consortium-3 (VARC-3) criteria [26], categorizing PVR as none/trace, mild, moderate, or severe. Given the reader's specialized expertise, a single-reader assessment was employed, with VARC-3 criteria serving to ensure standardization.

### 2.4 Procedural details

Three SE-THVs systems were implanted in this study: the Venus A-valve (Venus MedTech) with no sealing skirts [27], and other two valves with sealing skirts: The VitaFlow valve (MicroPort Medical) [28], and TaurusOne valve (Peijia Medical) [29]. Valve sizes ranging from 21 to 32 mm were selected based on CT-derived annular dimensions in accordance with manufacturer recommendations. All the procedure were performed through transfemoral access. Pre-dilation and post-dilation were performed at the operator’s discretion based on angiographic and echocardiographic findings during the procedure. Details of prosthetic valve technical characteristics and potential impact on PVR were shown in Supplementary Table 1.

### 2.5 Statistical analysis

Continuous variables are presented as medians with interquartile ranges (IQR) or as means ± standard deviations (SD), as appropriate. Categorical variables are expressed as frequencies and percentages. Group comparisons were performed using the chi-square test, Student’s *t*-test, or the Mann–Whitney *U* test, depending on data distribution and scale.

Univariate and multivariate logistic regression models (enter method) were used to identify independent predictors of mild/moderate PVR. Results are reported as odds ratios (OR) with corresponding 95 % confidence intervals (CI). To assess multicollinearity between aortic annular perimeter and THV perimeter oversizing, correlation analysis was conducted and variance inflation factors (VIF) were calculated.

The predictive performance of individual predictors and their combination was evaluated using receiver operating characteristic (ROC) curves, with area under the curve (AUC) values compared using the DeLong test. The integrated discrimination improvement (IDI) was employed to quantify the enhancement in predictive accuracy offered by the combined model. Additionally, the Brier score was calculated to assess the overall accuracy of the predictive models, with lower values indicating better predictive performance, and calibration curve was used to evaluate the conformity of the model.

Multivariate logistic regression identified aortic annulus perimeter and device landing zone calcific volume as two independent anatomical predictors. The optimal cut-off values were determined using Youden index (sensitivity + specificity - 1) to identify the thresholds that maximize discriminatory power. The derived thresholds were 1240.4 mm³ for calcific volume and 77.2 mm for annular perimeter. These thresholds were clinically adjusted to 77 mm and 1240 mm³ for practical application, and applied to stratify the entire cohort into three distinct risk groups: Group A (Low Risk): Calcific volume < 1240 mm³ and annular perimeter < 77 mm; Group B (Intermediate Risk): Calcific volume ≥ 1240 mm³ or annular perimeter ≥ 77 mm; Group C (High Risk): Calcific volume ≥ 1240 mm³ and annular perimeter ≥ 77 mm.

The primary outcome was the incidence of mild/moderate PVR across groups, with pairwise comparisons (A vs. B, B vs. C) performed to validate the graded increase in risk. Subgroup analyses were conducted to evaluate the effect of sealing skirts within each risk category.

Interobserver variability for imaging measurements was assessed using the intraclass correlation coefficient (ICC) and Bland–Altman analysis. All analyses were performed with MedCalc (v19.6.4) and R software (v3.4.2), and a two-sided *P* < 0.05 was considered statistically significant.

## 3 Results

### 3.1 Baseline characters

A total of 292 patients were included. Baseline demographics and clinical characteristics are summarized in Table 1. The cohort had a mean age of 67.9 ± 8.0 years, 56.5 % (165/292) were male, and 52.1 % (152/292), 24.0 % (70/292), 19.5 % (57/292), and 4.5 % (13/292) had none, trace, mild, and moderate PVR. Except for significantly higher proportion of male gender (71.4 % vs. 51.8 %, *P* = 0.004) and moderate-severe MR (25.7 % vs. 14.0 %, *P* = 0.022) in mild/moderate than none/trace PVR, the two groups had similar demographics, clinical characteristics, and echocardiographic data (*P* > 0.05).

**Table 1:** Baseline characteristics stratified by PVR.

Characters	Total (n = 292)	None/Trace (n = 222)	Mild/Moderate (n = 70)	*P*
**Demographics**				
Age (years)	67.9 ± 8.0	67.9 ± 8.2	68.0 ± 7.5	0.915
Male, n (%)	165 (56.5)	115 (51.8)	50 (71.4)	**0.004**
Body mass index (kg/m^2^)	24.0 ± 3.8	24.1 ± 4.1	23.3 ± 2.8	0.091
Body surface area (m^2^)	1.7 ± 0.2	1.7 ± 0.2	1.7 ± 0.2	0.621
**Clinical characteristics**				
Diabetes, n (%)	39 (13.4)	33 (14.9)	5 (7.1)	0.095
Hypertension, n (%)	100 (34.2)	81 (36.5)	19 (27.1)	0.152
Coronary artery disease, n (%)	72 (24.7)	51 (23.0)	21 (30.0)	0.235
Dyslipidemia, n (%)	17 (5.8)	14 (6.3)	3 (4.3)	0.529
Smoking, n (%)	67 (22.9)	51 (23.0)	16 (22.9)	0.984
EuroSCORE II	1.9 (1.2–3.4)	1.9 (1.2–3.2)	2.2 (1.4–4.0)	0.940
NYHA ≥ 3, n (%)	277 (94.9)	211 (95.0)	66 (94.3)	0.802
**Echocardiographic data**				
LVEF (%)	53.0 (42.0–57.0)	54.0 (42.0–58.0)	52.5 (42.0–55.0)	0.126
Peak aortic valve velocity, (cm/s)	455.6 ± 77.7	454.1 ± 73.7	460.3 ± 65.3	0.129
Mean aortic valve gradient, (mmHg)	51.0 ± 17.4	50.0 ± 16.6	54.2 ± 19.5	0.115
Aortic valve area, (cm^2^)	0.7 (0.6–0.9)	0.7 (0.6–0.9)	0.7 (0.5–0.8)	0.270
Moderate-severe AR, n (%)	56 (19.2)	40 (18.0)	16 (22.9)	0.371
Moderate-severe MR, n (%)	49 (16.8)	31 (14.0)	18 (25.7)	**0.022**
Moderate-severe TR, n (%)	19 (6.5)	13 (5.9)	6 (8.6)	0.422
**Computed tomography data**				
Bicuspid aortic valve, n (%)	166 (56.8)	125 (56.3)	41 (58.6)	0.739
Ascending aorta diameter, mm	39.6 (36.0–43.6)	39.7 (35.9–43.4)	39.4 (36.4–44.1)	0.410
Left coronary height, mm	12.9 (10.9–15.2)	12.6 (10.9–15.0)	13.3 (11.0–16.3)	0.386
Right coronary height, mm	14.0 (11.8–16.4)	13.8 (11.6–16.3)	14.3 (12.4–16.5)	0.391
Ellipticity index, %	76.4 (70.9–84.5)	77.0 (70.9–85.7)	74.8 (70.8–81.7)	0.215
Aortic annulus perimeter, mm	76.9 (71.0–83.9)	75.7 (69.3–81.9)	84.0 (78.0–87.1)	**< 0.001**
THV perimeter oversizing, %	8.1 ± 10.7	9.7 ± 10.4	3.0 ± 10.0	**< 0.001**
Device landing zone calcific volume, mm^3^	895.9 (391.1–1517.7)	750.7 (334.7–1324.0)	1443.8 (771.6–2265.0)	**< 0.001**
Leaflet calcium grade, n (%)				0.060
None-mild	89 (30.5)	74 (33.3)	15 (21.4)	
Mod-severe	203 (69.5)	148 (66.7)	55 (78.6)	
Annular calcium grade, n (%)				**0.044**
None-mild	172 (58.9)	138 (62.2)	34 (48.6)	
Mod-severe	120 (41.1)	84 (37.8)	36 (51.4)	
LVOT calcium grade, n (%)				**0.021**
None-mild	222 (76.0)	176 (60.3)	46 (65.7)	
Mod-severe	70 (24.0)	46 (15.8)	24 (34.3)	
**TAVR Procedure data**				
Pre-dilatation, n (%)	213 (72.9)	158 (71.2)	55 (78.6)	0.225
Post-dilatation, n (%)	105 (36.0)	74 (33.3)	31 (44.3)	0.083
THVs with sealing skirt, n (%)	117 (40.1)	97 (43.7)	20 (28.6)	**0.025**

Note: PVR = Paravalvular regurgitation; EuroSCORE II = European System for Cardiac Operative Risk Evaluation II; NYHA = New York Heart Association; TAVR = Transcatheter aortic valve replacement; THVs = Transcatheter heart valves; LVEF = left ventricular ejection fraction; AR = aortic regurgitation; MR = mitral regurgitation; TR = tricuspid regurgitation. LVOT = left ventricular outflow tract.

With regard to computed tomography data, the mild/moderate PVR group exhibited larger aortic annulus perimeter (84.0 vs. 75.7 mm, *P* < 0.001), higher device landing zone calcific volume (1443.8 vs. 750.7 mm³, *P* < 0.001), and smaller THV perimeter oversizing rate (3.0 ± 10.0 % vs. 9.7 ± 10.4 %, *P* < 0.001). Besides, the mild/moderate PVR group had significantly lower THVs with sealing skirt than none/trace PVR group (28.6 % vs. 43.7 %, *P* = 0.025). No significant difference was observed for other CT and procedure data (*P* > 0.05). Among the cohort, 95 and 71 patients had Sievers type 0 and type 1 bicuspid aortic valves, respectively. The incidence of mild/moderate PVR was similar between these groups: 26.3 % (25/95) in type 0 and 23.9 % (17/71) in type 1 (P = 0.729).

Regarding prosthesis type, 175 patients received the Venus A-Valve, 72 received the VitaFlow valve, and 45 received the TaurusOne valve. The incidence of mild/moderate PVR was 28.6 % (50/175), 18.1 % (13/72), and 15.6 % (7/45), respectively. No statistically significant difference in PVR incidence was observed among the three valve types (P = 0.076, chi-square test).

### 3.2 Independent predictors of PVR

Univariate logistic regression identified male gender (OR: 2.326, 95 % CI: 1.300 – 4.161, *P* = 0.004), moderate-severe MR (OR: 2.133, 95 %CI: 1.106 – 4.113), aortic annulus perimeter (OR: 1.094, 95 % CI: 1.058–1.132, *P* < 0.001), THVs perimeter oversizing (OR: 0.935, 95 % CI:0.908 – 0.963), device landing zone calcific volume (OR: 1.009 per 10 mm³, 95 % CI: 1.005 – 1.012, *P* < 0.001), annular calcium grade (OR: 1.740, 95 % CI: 1.012 – 2.990, *P* = 0.045), LVOT calcium grade (OR: 1.996, 95 % CI: 1.106 – 3.604, *P* = 0.022), and THVs with sealing skirt (OR: 0.516, 95 %CI: 0.288 – 0.923) as potential predictors of mild/moderate PVR (Table 2). Correlation coefficient between aortic annulus perimeter and THV perimeter oversizing was −0.638 (95 % CI: −0.701 – −0.564), *P* < 0.001 (Supplementary Figure 3), VIF between aortic annulus perimeter and THV perimeter oversizing was 1.685 (below 3), indicating although significant negative correlation, the multicollinearity was not a concern.

**Table 2:** Univariate and multivariate logistic regression analysis of potential risk predictors for PVR.

	Univariate		Multivariate	
	Odds Ratio	*P* value	Odds Ratio	*P* value
**Demographics**				
Age, per year	1.001 (0.969 – 1.036)	0.915		
Male	2.326 (1.300 – 4.161)	**0.004**	0.790 (0.357 – 1.747)	0.560
Body mass index, per kg/m^2^	0.937 (0.870 – 1.011)	0.092		
Body surface area, per m^2^	1.454 (0.332 – 6.360)	0.619		
**Clinical characteristics**				
Diabetes	0.441 (0.165 – 1.176)	0.102		
Hypertension	0.649 (0.358 – 1.174)	0.153		
Coronary artery disease	1.437 (0.789 – 2.616)	0.236		
Dyslipidemia	0.665 (0.186 – 2.386)	0.532		
Smoking	0.994 (0.524 – 1.883)	0.984		
EuroSCORE II	0.998 (0.940 – 1.059)	0.9491		
NYHA ≥ 3	0.860 (0.265 – 2.792)	0.802		
**Echocardiographic data**				
LVEF, per %	0.983 (0.961 – 1.004)	0.116		
Peak aortic valve velocity, per cm/s	1.001 (0.998 – 1.029)	0.227		
Mean aortic valve gradient, per mmHg	1.013 (1.000 – 1.026)	0.190		
Aortic valve area, per cm^2^	0.631 (0.221 – 1.803)	0.390		
Moderate-severe AR	1.348 (0.701 – 2.594)	0.371		
Moderate-severe MR	2.133 (1.106 – 4.113)	**0.024**	2.042 (0.973 – 4.288)	0.059
Moderate-severe TR	1.507 (0.551 – 4.126)	0.425		
**Computed tomography data**				
Bicuspid aortic valve	0.912 (0.529 – 1.571)	0.739		
Ascending aorta diameter, per mm	1.019 (0.975 – 1.066)	0.397		
Left coronary height, per mm	1.034 (0.964 – 1.110)	0.352		
Right coronary height, per mm	1.026 (0.960 – 1.097)	0.455		
Ellipticity index, per %	0.990 (0.972 – 1.009)	0.292		
Aortic annulus perimeter, per mm	1.094 (1.058 – 1.132)	**< 0.001**	1.067 (1.013 – 1.123)	**0.015**
THV perimeter oversizing, per %	0.935 (0.908 – 0.963)	**< 0.001**	0.977 (0.937 – 1.019)	0.278
Device landing zone calcific volume, per 10 mm^3^	1.009 (1.005 – 1.012)	**< 0.001**	1.006 (1.001 – 1.011)	**0.025**
Leaflet calcium grade	1.833 (0.971 – 3.461)	0.062		
Annular calcium grade	1.740 (1.012 – 2.990)	**0.045**	1.748 (0.944 – 3.239)	0.076
LVOT calcium grade	1.996 (1.106 – 3.604)	**0.022**	0.888 (0.402 – 1.962)	0.770
**Procedure data**				
THVs with sealing skirt	0.516 (0.288 – 0.923)	**0.026**	0.412 (0.209 – 0.811)	**0.010**
Pre-dilatation	1.485 (0.783 – 2.818)	0.226		
Post-dilatation	1.630 (0.937 – 2.836)	0.083		

Note: PVR = Paravalvular regurgitation; EuroSCORE II = European System for Cardiac Operative Risk Evaluation II; NYHA = New York Heart Association; TAVR = Transcatheter aortic valve replacement; THV =Transcatheter heart valve; LVEF = left ventricular ejection fraction; AR = aortic regurgitation; MR = mitral regurgitation; TR = tricuspid regurgitation; LVOT = left ventricular outflow tract.

In multivariate analysis, aortic annulus perimeter (OR: 1.067, 95 % CI: 1.013 – 1.123, *P* = 0.015), device landing zone calcific volume (OR: 1.006 per 10 mm³, 95 % CI: 1.001 – 1.011, *P* = 0.025), and THVs with sealing skirt (OR:0.412, 95 %CI: 0.209 – 0.811, *P* = 0.010) remained independent predictors, while the significance of gender, moderate-severe MR, THV perimeter oversizing, annular and LVOT calcium grade disappeared (*P* > 0.05). (Table 2).

### 3.3 Risk stratification by annular perimeter and device landing zone calcification

**Fig. 1:**
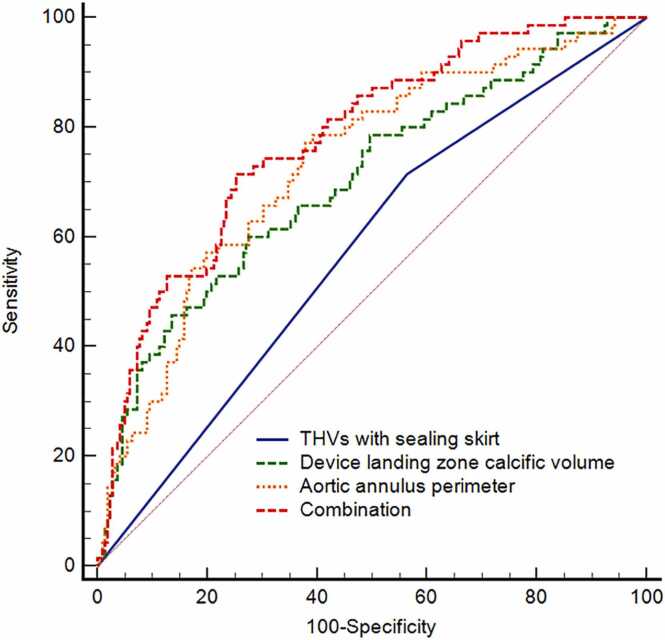
ROC analysis of parameters to predict mild/moderate PVR after TAVR. The AUCs of THVs with sealing skirt, device landing zone calcific volume, and aortic annulus perimeter were 0.576 (0.517 – 0.633), 0.702 (0.646 – 0.754) and 0.733 (0.678 – 0.783), significantly lower than their combination of 0.779 (0.727 – 0.826) (P < 0.001, 0.007, and 0.036). The optimal cutoff value of device landing zone calcific volume was > 1240.4 mm^3^, with sensitivity and specificity of 60.0 % and 72.5 %; The optimal cutoff value of aortic annulus perimeter was > 77.2 mm, with sensitivity and specificity of 78.6 % and 60.8 %. Note: ROC = receiver operator characteristic curve; PVR = paravalvular regurgitation; AUC = area under the curve; THVs = transcatheter heart valves.

**Fig. 2:**
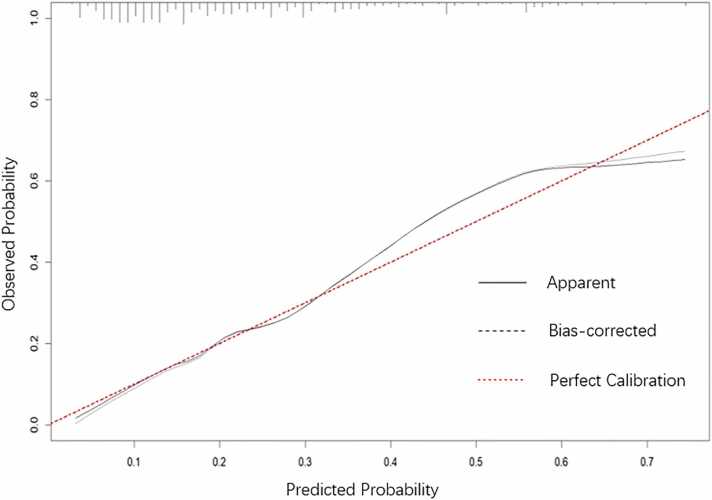
Calibration plot of the combination of THVs with sealing skirt, device landing zone calcific volume, and aortic annulus perimeter for mild/moderate PVR prediction. The figure illustrates the agreement between the predicted probability of mild/moderate PVR by the dual-parameter model (x-axis) and the observed actual frequency (y-axis). The close proximity of the bias-corrected curve to the line of perfect calibration indicates good model calibration.

**Fig. 3:**
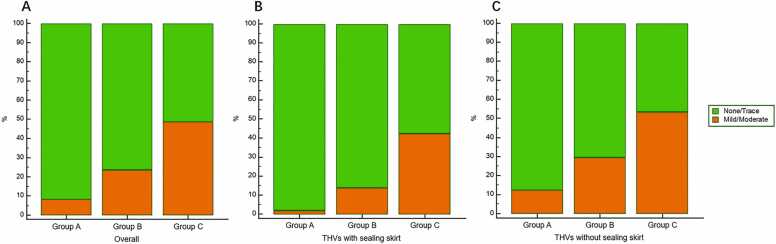
Incidence of mild/moderate PVR stratified by device landing zone calcific volume and annular perimeter. Overall (3 A): Incidence in Group A, B, and C were 8.4 % (10/119), 23.7 % (23/97), and 48.7 % (37/76). (*P* for trend < 0.001; Group A vs. B, *P* = 0.002; Group B vs. C, *P* < 0.001). THVs with sealing skirt (3B): Incidence of 2.1 % (1/48), 13.9 % (5/36), and 42.4 % (14/33) in Groups A, B, and C (*P* for trend < 0.001; Group A vs. B, *P* = 0.039; Group B vs. C, *P* = 0.009). THVs without sealing skirt (3 C): Incidence of 12.7 % (9/71), 29.5 % (18/61), and 53.5 % (23/43) in Groups A, B, and C (*P* for trend < 0.001; Group A vs. B, *P* = 0.017; Group B vs. C, *P* = 0.014). Note: Group definitions: group A (device landing zone calcific volume <1240 mm³ and aortic annulus perimeter <77 mm); group B (either parameter elevated); group C (both elevated). PVR = paravalvular regurgitation; THVs = transcatheter heart valves.

The combined model yielded an AUC of 0.779 (95 % CI: 0.727–0.826), which was significantly higher than that of aortic annular perimeter alone (AUC = 0.733, 95 % CI: 0.678–0.783; P = 0.036), device landing zone calcific volume alone (AUC = 0.702, 95 % CI: 0.646–0.754; P = 0.007), and THVs with sealing skirt alone (AUC = 0.576, 95 % CI: 0.517–0.633; P < 0.001) in predicting mild/moderate PVR (Fig. 1). The integrated discrimination improvement (IDI) also showed significant enhancements of 5.4 % (95 % CI: 2.5–8.6; P < 0.001), 6.7 % (95 % CI: 3.5–10.3; P < 0.001), and 15.7 % (95 % CI: 11.4–20.6; P < 0.001), respectively. Furthermore, the combined model achieved the lowest Brier score of 0.151. Furthermore, the calibration plot demonstrated good agreement between the model-predicted probabilities and the observed outcomes across the entire range of predicted risk (Fig. 2). Sensitivity, specificity, and accuracy for each parameter are presented in Table 3. Using Youden index analysis, optimal CT-derived cutoffs were established at 77.2 mm for aortic annular perimeter and 1240.4 mm³ for device landing zone calcific volume, and clinically adjusted to 1240 mm³ and 77 mm for practical use. Stratification into three groups revealed a stepwise increase in PVR incidence: Group A (aortic valve calcific volume < 1240 mm^3^ and aortic annulus perimeter < 77 mm, n = 119): 8.4 % (10/119); Group B (either parameter elevated, n = 97): 23.7 % (23/97); Group C (both parameters elevated, n = 77): 48.7 % (37/76). Trend analysis showed a significant increase in PVR risk across groups (*P* < 0.001), with pairwise comparisons confirming differences between Group A vs. B (*P* = 0.003) and Group B vs. C (*P* < 0.001). (Fig. 3).

**Table 3:** Diagnostic performance and discrimination improvement across single parameters and their combination for predicting mild/moderate PVR.

Parameters	Sensitivity (95 % CI)	Specificity (95 % CI)	Accuracy (95 % CI)	Brier score	IDI (95 % CI)	IDI P value
Combination	71.4 (59.4 – 81.6)	74.3 (68.1 – 79.9)	73.6 (68.3 – 78.4)	0.151	-	
Aortic annulus perimeter	78.6 (67.1 – 87.5)	59.5 (52.7 – 66.0)	64.0 (58.4 – 69.3)	0.160	5.4 (2.5 – 8.6) *	< 0.001 *
Device landing zone calcific volume	60.0 (47.6 – 71.5)	72.1 (65.7 – 77.9)	69.2 (63.7 – 74.2)	0.163	6.7 (3.5 – 10.3) †	< 0.001 †
THVs with sealing skirt	71.4 (59.4 – 81.6)	43.7 (37.1 – 50.5)	50.3 (44.6 – 56.0)	0.179	15.7 (11.4 – 20.6) #	< 0.001 #

Note: PVR = Paravalvular regurgitation; CI = confidence interval; IDI = integrated discrimination improvement; THVs = Transcatheter heart valves. * Aortic annulus perimeter vs. combination; † Device landing zone calcific volume vs. combination; # THVs with sealing skirt vs. Combination.

### 3.4 Impact of sealing skirts on the PVR

Besides, when dichotomized the patients into THVs with and without sealing skirts, the trends still remained: Among THVs with sealing skirts, incidence of mild/moderate PVR in group A, B, and C were 2.1 % (1/48), 13.9 % (5/36), and 42.4 % (14/33), differences between Group A vs. B and Group B vs. C were significant (*P* = 0.039 and 0.009); Among THVs without sealing skirts, incidence of mild/moderate PVR in group A, B, and C were 12.7 % (9/71), 29.5 % (18/61), and 53.5 % (23/43), differences between Group A vs. B and Group B vs. C were also significant (*P* = 0.017 and 0.014).(Fig. 3).

Furthermore, among Group A, THVs with sealing skirt had a significantly lower incidence of mild/moderate PVR (2.1 % vs. 12.7 %, *P* = 0.042); Among group B and C, THVs with sealing skirt had lower but no significantly difference incidence of mild/moderate PVR (Group B: 13.9 % vs. 29.5 %, *P* = 0.082; Group C: 42.4 % vs. 53.5 %, *P* = 0.342) (Table 4).

**Table 4:** Impact of sealing skirt on PVR among different risk groups.

	Group A (n = 119)			Group B (n = 97)			Group C (n = 76)		
	Sealing (n = 48)	No sealing (n = 71)	*P*	Sealing (n = 36)	No sealing (n = 61)	*P*	Sealing (m = 33)	No sealing (n = 43)	*P*
None/Trace, n (%)	47 (97.9)	62 (87.3)	0.042	31 (86.1)	43 (70.5)	0.082	19 (57.6)	20 (46.5)	0.342
Mild/Moderate, n (%)	1 (2.1)	9 (12.7)		5 (13.9)	18 (29.5)		14 (42.4)	23 (53.5)	

Group definitions: A (calcification <1240 mm³ and perimeter <77 mm); B (either parameter elevated); C (both elevated). Note: PVR = paravalvular regurgitation.

### 3.5 Reproducibility of aortic annulus perimeter and device landing zone calcific volume assessment

**Fig. 4:**
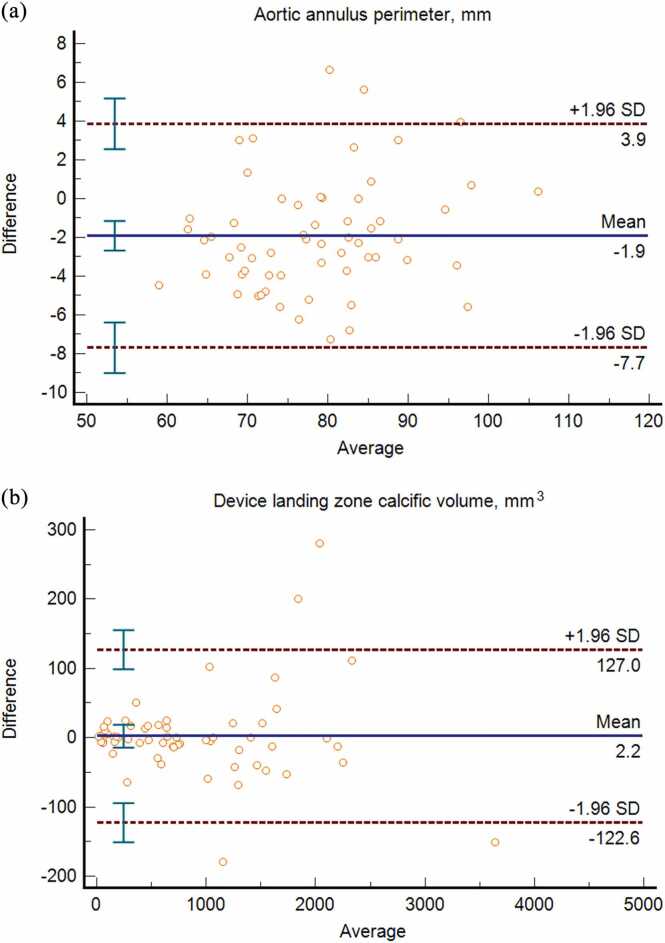
Bland–Altman analysis for aortic annulus perimeter (4 A) and device landing zone calcific volume (4B) and measurements.

The interobserver agreement for the measurements of the aortic annulus perimeter was high (ICC=0.969, 95 % CI:0.906–0.986); Bland-Altman analysis showed a mean bias of –1.9 mm, as presented in Fig. 4.

Concurrently, the interobserver repeatability of measuring the calcific volumes within the device landing zone was high (ICC=0.998, 95 % CI:0.997–0.999); Bland-Altman analysis showed minimal bias (mean difference=2.2 mm³), as illustrated in Fig. 4**.**

## 4 Discussion

The findings of this study reveal that the combined assessment of device landing zone calcification and aortic annular perimeter significantly enhances the ability to predict PVR following SE-TAVR. Our dual-parameter model demonstrated a stratified predictive performance, with patients exhibiting both elevated device landing zone calcific volume (≥1240 mm³) and enlarged annular perimeter (≥77 mm) facing a markedly higher incidence of mild/moderate PVR (48.7 %) compared to those with only one (23.7 %) or neither (8.4 %) risk factor. Notably, the protective effect of sealing skirts was most pronounced in patients with lower calcific burden and smaller annular dimensions, underscoring the importance of tailored device selection based on pre-procedural imaging assessment. These results provide a compelling rationale for integrating quantitative calcification analysis and annular sizing into clinical decision-making to optimize outcomes in SE-TAVR.

Our findings align with previous studies identifying device landing zone calcification and annular dimensions as important determinants of PVR following TAVR [5], [12], [13], [14], [15], [16], [17]. However, this study makes three significant advances: First, by focusing exclusively on SE-TAVR recipients, we elucidate the unique biomechanical interactions between SE-THV design characteristics and calcification/annular anatomy. Second, our application of Gaussian mixture modeling for calcification quantification overcomes the limitations of traditional qualitative assessments or heterogeneous threshold methods [19], [20], providing high measurement accuracy and reproducibility. Third, through rigorous ROC analysis, we established clinically actionable cutoff values (device landing zone calcific volume ≥1240 mm³; annular perimeter ≥77 mm) that offer objective standards for preprocedural risk stratification. These methodological innovations collectively enabled our predictive model to achieve moderate-to-good discriminative performance discriminative performance (AUC=0.779), significantly outperforming single-parameter predictions, with good calibration.

Notably, our findings contrast with a previous meta-analysis [11], which reported for THVs with sealing skirt, the amount of calcium has no significant effect on the incidence of PVL, potentially attributable to the meta-analysis combined data from both SE- and BE-THV designs, and did not account for the critical interaction between calcification and annular dimensions, and their analysis included first-generation valves with primitive sealing technology, whereas our cohort exclusively evaluated contemporary SE-THVs with advanced skirt designs (VitaFlow/TaurusOne).

The mechanistic basis for our findings likely involves two key pathways: First, calcific deposits create rigid barriers that hinder stent expansion and circumferential sealing, particularly at the annulus and interleaflet triangles [12], [17]. Second, calcification reduces annular compliance, compromising the passive radial force-dependent apposition of SE-THVs, which lack the controlled expansion mechanism of BE-THVs [10], [11]. These effects are exacerbated in large annuli, where the combined risk profile of increased annular diameter and focal stress concentration on calcified segments explains the disproportionately high PVR rate in Group C. Here, calcification effectively enlarges the functional annulus while disrupting uniform stent expansion.

Clinically, these findings advocate for a tailored approach to SE-TAVR. For patients with combined high device landing zone calcification burden (≥1240 mm³) and large aortic annular perimeter (≥77 mm, Group C), BE-THVs may be preferred due to their superior radial force for calcified and dilated anatomies [10], [16], while SE-THVs with advanced sealing skirts could be prioritized for lower-risk anatomies (Groups A and B) where their protective effect is most pronounced. The loss of oversizing’s significance in multivariate analysis underscores a key clinical insight: preprocedural assessment of annular anatomy (not just post-hoc oversizing calculations) should guide PVR risk stratification. Oversizing is a practical tool to align THV size with annular dimensions, but it does not provide independent information beyond annular perimeter—confirming that anatomical factors are more clinically actionable for SE-TAVR planning. Intra-procedurally, meticulous techniques—such as slow, controlled deployment and targeted post-dilation of calcified segments—may optimize stent apposition in high-risk cases, though caution is needed to avoid complications [12], [30]. Future SE-THVs designs should incorporate adaptive radial force profiles and calcification-conforming sealing mechanisms to address the risks associated with specific anatomical profiles (namely, high calcific burden and large annular perimeter) identified in this study. This stratified approach, guided by quantitative MDCT assessment, may refine valve selection and procedural strategies to improve outcomes.

This study has several limitations that should be acknowledged. First, its single-center, retrospective design may introduce selection bias and limit generalizability; although the standardized imaging and procedural protocols across all patients may partially mitigate heterogeneity. Second, the short follow-up period (predischarge PVR assessment) precludes evaluation of long-term valve durability and the potential progression of PVR severity over time, future studies should include extended follow-up to address this gap. Third, despite rigorous multivariate adjustments, unmeasured confounders—such as virtual raphe ring perimeter could influence PVR outcomes [17], were not systematically analyzed. Fourth, the study exclusively included Chinese patients, and the predominance of bicuspid aortic valves (56.8 %) may limit extrapolation to Western populations with distinct valvular anatomies and calcification patterns. Future prospective studies integrating advanced calcium distribution metrics and diverse populations are warranted.

## 5 Conclusions

In conclusion, this study demonstrates that the combined presence of device landing zone calcification (≥1240 mm³) and enlarged annular perimeter (≥77 mm) significantly increases PVR risk after SE-TAVR. The proposed dual-threshold model provides a clinically actionable tool for pre-procedural risk stratification.

## CRediT authorship contribution statement

**Yao Zhao:** Formal analysis, Data curation. **Jian Yang:** Project administration. **Minwen Zheng:** Writing – review & editing, Supervision, Funding acquisition. **Jun Shu:** Writing – original draft, Methodology, Data curation, Conceptualization. **Didi Wen:** Funding acquisition, Formal analysis. **Jingji Xu:** Writing – original draft, Software. **Yu Mao:** Validation, Resources, Investigation. **Hui Ma:** Visualization, Data curation. **Jing Zhang:** Validation, Software.

## Ethics

The ethics committee of the Xijing Hospital, Fourth Military Medical University approved this study (IRB No. KY20253499–1), Informed consent was waived for retrospective data analysis.

## Funding

This study was supported by the 10.13039/501100001809National Natural Science Foundation of China (grant numbers 82202149 and 82371953).

## Declaration of Competing Interest

The authors declare that they have no known competing financial interests or personal relationships that could have appeared to influence the work reported in this paper.

## Footnotes

Appendix A Supplementary data associated with this article can be found in the online version at doi:10.1016/j.ejro.2025.100719.
