# Supplementary material for: Dual-parameter risk stratification based on device landing zone calcification and aortic annular perimeter for paravalvular regurgitation after self-expanding TAVR
Source: Eur J Radiol Open. 2025 Dec 10;16:100719. doi: 10.1016/j.ejro.2025.100719 (PMC12752758; doi:10.1016/j.ejro.2025.100719)
Supplement: Supplementary file 1 — Supplementary material [file mmc1.docx]

**Supplement**


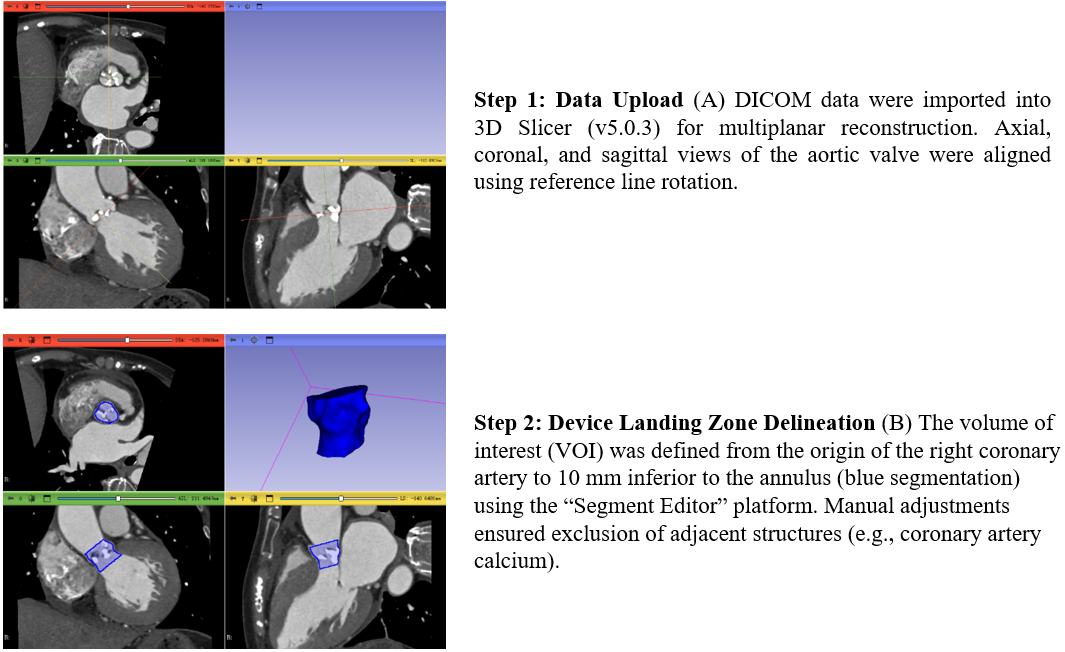


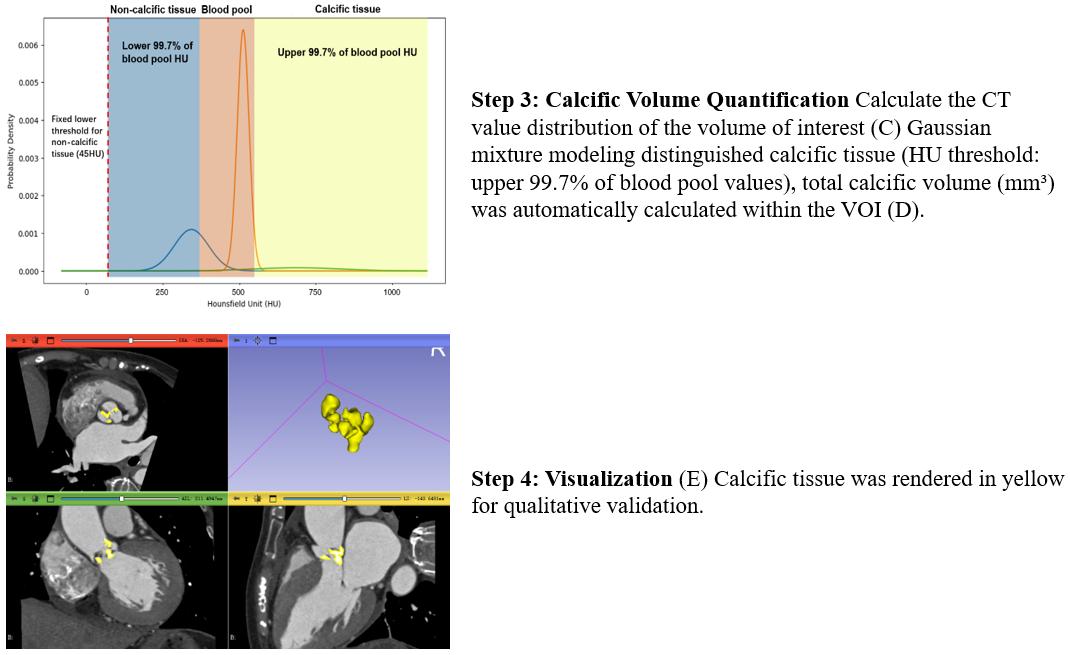


**Supplementary Figure 1.** Detailed process of device landing zone calcific volume analysis.


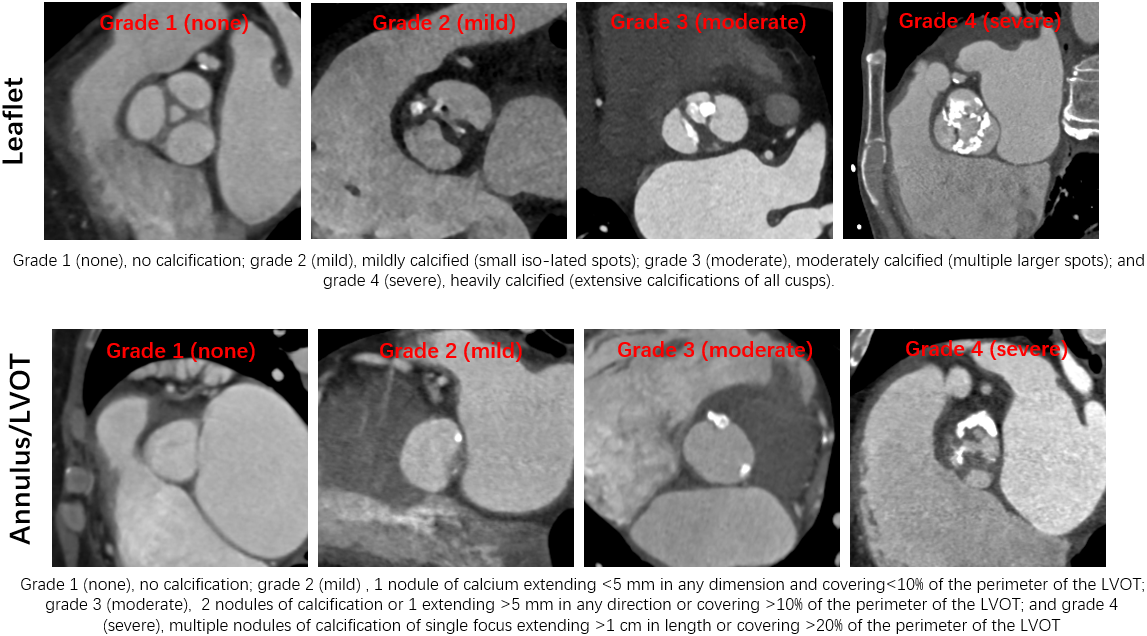


**Supplementary Figure 2** Semiquantitative evaluation of aortic valve leaflet, annulus and left ventricular outflow tract calcifications [1].

**
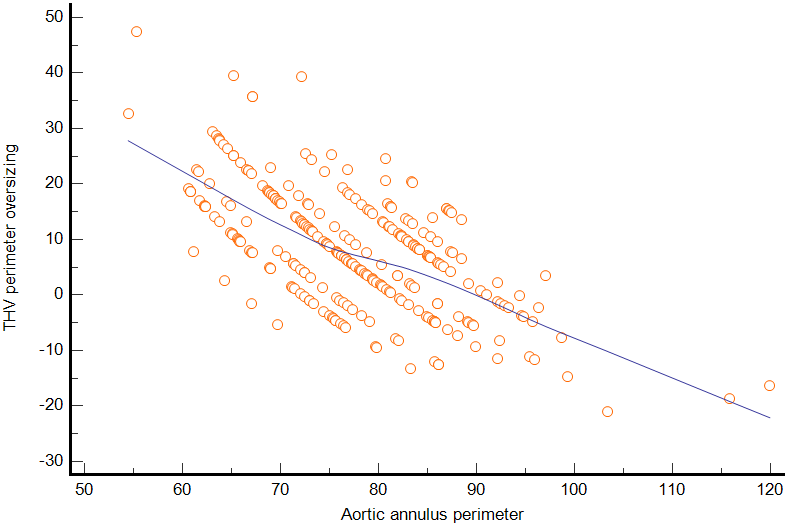
**

**Supplementary Figure 3** Correlation analysis between Aortic annulus perimeter and THV perimeter oversizing. The correlation coefficient was -0.638 (95% CI: -0.701 – -0.564), *P* < 0.001.

**Supplementary Table 1** Technical Characteristics and PVR Impact of Three SE-THVs Used in the Study.

| Characteristic | Venus A-Valve  (Venus MedTech) | VitaFlow Valve  (MicroPort Medical) | TaurusOne Valve  (Peijia Medical) |
| --- | --- | --- | --- |
| Stent Design | Nitinol self-expanding stent; open double-helix configuration (offers excellent radial support and compliance for native annulus adaptation). | Nitinol self-expanding stent; densely configured cellular structure (enhances radial force for stable implantation). | Nitinol self-expanding stent; unique "double-row buckle" design (improves anchoring force and sealing capabilities). |
| Leaflet Material | Anti-calcification-treated porcine pericardium (ensures durability and biocompatibility). | Anti-calcification-treated bovine pericardium (provides favorable mechanical properties and long-term performance). | Anti-calcification-treated porcine pericardium (guarantees reliability and functional stability). |
| Sealing Design | No dedicated sealing skirt; relies on stent radial force and valve-native annulus apposition for sealing. | External polyethylene terephthalate (PET) sealing skirt (covers lower one-third of the stent to enhance sealing). | Double-layer PET sealing skirt (positioned at middle and lower stent segments; extends sealing area). |
| Implantation Method | Transfemoral approach; fully retrievable and repositionable (improves implantation precision). | Transfemoral approach; partially retrievable (allows intraoperative adjustments). | Transfemoral approach; fully retrievable and repositionable (enables precise placement in complex anatomies). |
| Potential Impact on PVR | Higher susceptibility to paravalvular leakage (no active sealing mechanism), especially in cases of severe calcification or large aortic annulus. | Effective at sealing small paravalvular leaks; suitable for patients with moderate calcification and appropriately sized annulus. | Superior sealing performance in high-risk anatomies (e.g., uneven calcification, slightly enlarged annulus) due to dual-layer skirt. |

**Reference**

[1] Barbanti M, Yang TH, Rodès Cabau J, et al. Anatomical and procedural features associated with aortic root rupture during balloon-expandable transcatheter aortic valve replacement. Circulation. 2013 Jul 16;128(3):244-53.
